# Real-time computer-based visual feedback improves visual acuity in downbeat nystagmus – a pilot study

**DOI:** 10.1186/s12984-015-0109-2

**Published:** 2016-01-04

**Authors:** Julian Teufel, S. Bardins, Rainer Spiegel, O. Kremmyda, E. Schneider, M. Strupp, R. Kalla

**Affiliations:** Department of Neurology and German Center for Vertigo and Balance Disorders, Ludwig-Maximilans-University Munich, Marchioninistr. 15, 81377 Munich, Germany; Division of Internal Medicine, Basel University Hospital, Am Petersgraben 4, 4031 Basel, Switzerland; Institute of Medical Technology, Brandenburg University of Technology Cottbus-Senftenberg, Großenhainer Str. 57, 01968 Senftenberg, Germany; Department of Neurology Inselspital, Bern University Hospital, Freiburgstrasse 4, 3010 Bern, Switzerland

**Keywords:** Downbeat nystagmus, Visual acuity, Eye tracking, Video oculography, Computer-based visual feedback

## Abstract

**Background:**

Patients with downbeat nystagmus syndrome suffer from oscillopsia, which leads to an unstable visual perception and therefore impaired visual acuity. The aim of this study was to use real-time computer-based visual feedback to compensate for the destabilizing slow phase eye movements.

**Methods:**

The patients were sitting in front of a computer screen with the head fixed on a chin rest. The eye movements were recorded by an eye tracking system (EyeSeeCam®). We tested the visual acuity with a fixed Landolt C (static) and during real-time feedback driven condition (dynamic) in gaze straight ahead and (20°) sideward gaze. In the dynamic condition, the Landolt C moved according to the slow phase eye velocity of the downbeat nystagmus. The Shapiro-Wilk test was used to test for normal distribution and one-way ANOVA for comparison.

**Results:**

Ten patients with downbeat nystagmus were included in the study. Median age was 76 years and the median duration of symptoms was 6.3 years (SD +/- 3.1y). The mean slow phase velocity was moderate during gaze straight ahead (1.44°/s, SD +/- 1.18°/s) and increased significantly in sideward gaze (mean left 3.36°/s; right 3.58°/s). In gaze straight ahead, we found no difference between the static and feedback driven condition. In sideward gaze, visual acuity improved in five out of ten subjects during the feedback-driven condition (*p* = 0.043).

**Conclusions:**

This study provides proof of concept that non-invasive real-time computer-based visual feedback compensates for the SPV in DBN. Therefore, real-time visual feedback may be a promising aid for patients suffering from oscillopsia and impaired text reading on screen. Recent technological advances in the area of virtual reality displays might soon render this approach feasible in fully mobile settings.

## Background

Many patients with nystagmus (involuntary eye movements) suffer from blurred vision, unstable visual perception and decreased visual acuity (VA), which leads to a decreased quality of life. Amongst different forms of nystagmus, the downbeat nystagmus syndrome (DBN) is a frequent central type fixation nystagmus [1]. Patients predominantly suffer from balance disorders and oscillopsia. The latter results in a decrease of VA, in particular when looking downward during reading or to the right or left [2]. One cycle of a nystagmus consists of a slow phase and a saccade (fast phase), whereby the slow phase velocity (SPV = deg/s) generally is a measurement for the intensity of the nystagmus. While the slow phase of DBN induces oscillopsia, the fast phase of DBN does not induce oscillopsia, as visual perception is suppressed during the corrective downward saccade [3].

DBN intensity can be reduced in slightly over 50 % of patients [4–7] by aminopyridines, but many patients do not respond to drug therapy. Furthermore, patients may not tolerate the medical treatment or they still suffer from an impaired VA including text reading. Hence, there is reason to search for other treatment principles and assistive technology may be one of the principles helping to improve VA.

In this study, we investigated the effects of real-time visual feedback on the improvement of text reading measured by an increase in VA. Using a computer-assisted device (gaze-contingent-display) and an infrared video oculography system (EyeSeeCam®) [8], we created on a computer screen a real-time feedback image of the visual field, which was controlled by the slow phase velocity of DBN. A similar approach has been conducted before by using a magnetic search coil or an optical device [9, 10].

For the first time however, our aim was to stabilize the retinal image without the use of a magnetic search coil or an optical device. Therefore, we adopted a thoroughly different approach, without the need of any invasive procedures (coils or contact lenses) using a basically mobile set-up, though limited to a computer screen.

## Methods

Patients with DBN were included in this prospective study regardless of the etiology or the duration of visual symptoms (for clinical detail see Table 1). All patients gave their informed consent for participation in the study. The examination was performed in accordance with the Helsinki II Declaration and approved by the ethics committee of the Ludwig-Maximilians University Medical Faculty (No. 082/03).

**Table 1:** Etiology of the DBN syndrome (idiopathic cerebellar syndrome; cerebellar atrophy; CANVAS), duration of visual symptoms and visual acuity as measured by orthoptic exam (Snellen chart, CC “cum correctione”) as well as SPV in gaze straight ahead (Landolt C, SC “sin correctione”)

Age/sex	Etiology	Duration of symptoms (years)	Visual acuity Snellen chart	SPV in center gaze in °/s
72/m	idiopathic	10	0.6	2.5
65/f	idiopathic	3	1	1.47
82/f	CANVAS	4	0.75	0.1
79/m	idiopathic	13	0.6	1.0
75/f	CANVAS	7	0.25	3.0
80/m	idiopathic	7	0.75	3.5
55/m	CANVAS	3	0.8	0.06
59/f	idiopathic	6	0.8	1.0
78/f	atrophy	7	1	0.8
77/f	idiopathic	3	0.58	1.0
*Mean*	-	6.3	0.71	1.44
*SD*	-	+/-3.13	+/-0.21	+/-1.18

**Fig. 1:**
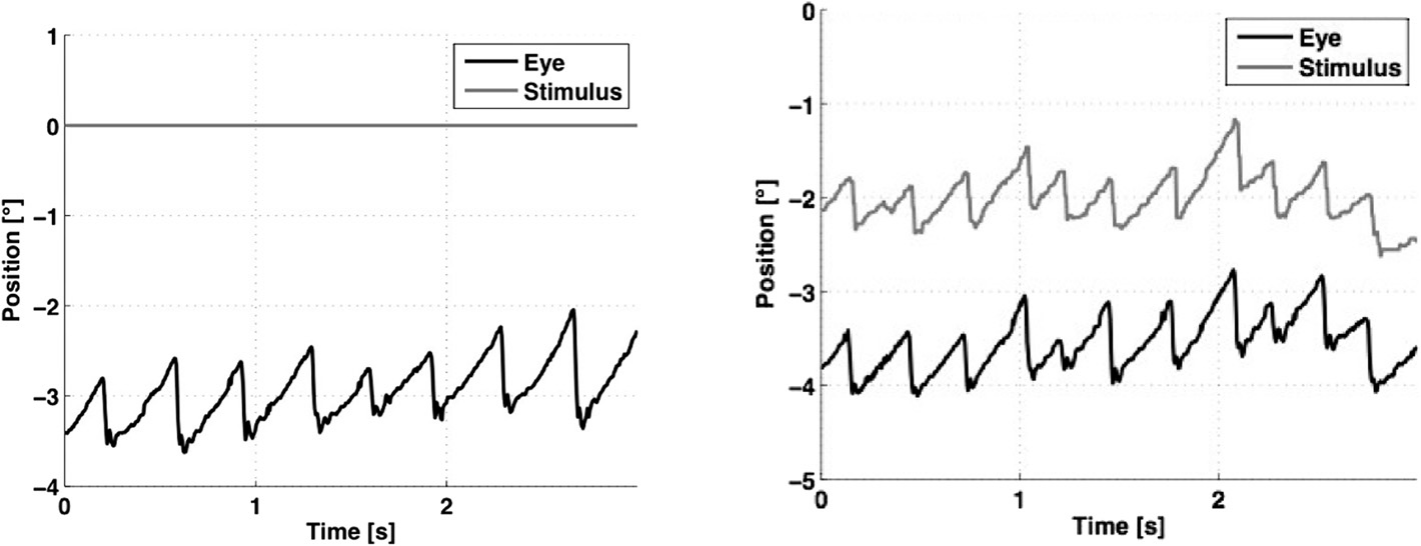
Vertical eye position (black line indicates vertical eye movements, gray line showing position of the Landolt C) in the static (left) and feedback-driven condition (right)

VA was measured by a standard orthoptic exam (Snellen chart, CC “cum correctione”) and using the EyeSeeCam® system during a static condition and dynamic feedback condition (Fig. 1; Landolt C, SC “sin correctione”). Each patient was sitting upright at a distance of 55 cm in front of a monitor with the head fixed on a chin rest. The patient’s eyes were detected by an eye tracking system consisting of one infrared camera (EyeSeeCam®) [8]. The eye was illuminated by an infrared light source integrated into the camera. A translucent hot mirror in front of each eye reflected only the infrared light in the direction of the camera. The camera was running at a sampling rate of 220 Hz. The eye position was measured with an accuracy of 0.5°. Visual stimulation was performed on an LCD monitor (BenQ, latency 2 ms, horizontal sync 120 Hz). The calibration was done presenting a center dot and four dots at 8.5° each left, right, up and down. We calculated clusters of the eye positions at each out of 5 fixation points (center, left, right, up, down). One cluster consisted of several slow and quick phases. Therefore, the center of the cluster was found approximately in the middle of the slow phase traces.

VA was measured in multiple trials based on the correct identification of the Landolt C optotype orientation. The Landolt C was designed according to standard guidelines (NAS-NRC, 1980) [11]. In each trial, one Landolt C was shown 6 times in different orientations. The size of the symbol on the initial trial was equivalent to a VA of 0.1. Each following trial the size of the Landolt C was decreased. The subjects’ results were entered manually by the experimenter. Each sequence was terminated after the participant erred on 2 consecutive judgements. The size of the Landolt C from the previous correct trial was used for determination of VA. The Landolt Cs were presented in two different conditions: static and feedback-driven. In each condition VA was tested in three positions relative to the head position: center, 20° to the left and 20° to the right. During the feedback-driven condition, the Landolt C was displayed in real-time movement with the same velocity as the upward drift of the eyes (=slow phase velocity; SPV). There was a latency of 20 ms between the onset of the upward slow phase and the onset of the movement of the Landolt C. In order to limit the ocular offset caused by a retinal error and small head movements, the Landolt C was shown in a predefined area (diameter 7.5°). The Landolt C position was automatically reset to the center point, in case it left the predefined area.

The eye movement velocity was calculated using a numerical three-point differentiation of the eye position and a Gaussian low-pass filtering with a corner frequency of 30Hz. The high-frequency velocity peaks of the nystagmus’ quick phases, saccades and blink artifacts were removed from the eye velocity using an absolute acceleration threshold of 700°/s^2 and a subsequent floating median filter with a time window of 0.5 s. Finally, SPV was calculated as the median during the presentation of the stimulus on a predefined position.

Statistical analysis was performed using SPSS (V 22, IBM Corp.). We applied the Shapiro-Wilk test to determine, whether the slow phase nystagmus velocity data were normally distributed. One-way ANOVA was used for comparison.

## Results

Ten Patients with DBN (4 male) with different etiologies were included in the study (cerebellar atrophy (*n* = 1), CANVAS (CANVAS = cerebellar ataxia, neuropathy, vestibular areflexia syndrome; *n* = 3), idiopathic (*n* = 6)). Median age was 76 years (SD +/- 8.9 y) and the mean duration of symptoms was 6.3 years (SD +/- 3.1 y; range 3-13 y). The results of the VA measurement by orthoptic exam (Snellen chart) and the static/feedback conditions (Landolt C) are listed in Table 1. As two patients had a visual acuity of 1, all patients were measured without eyeglasses (SC) to detect differences in both conditions.

**Fig. 2:**
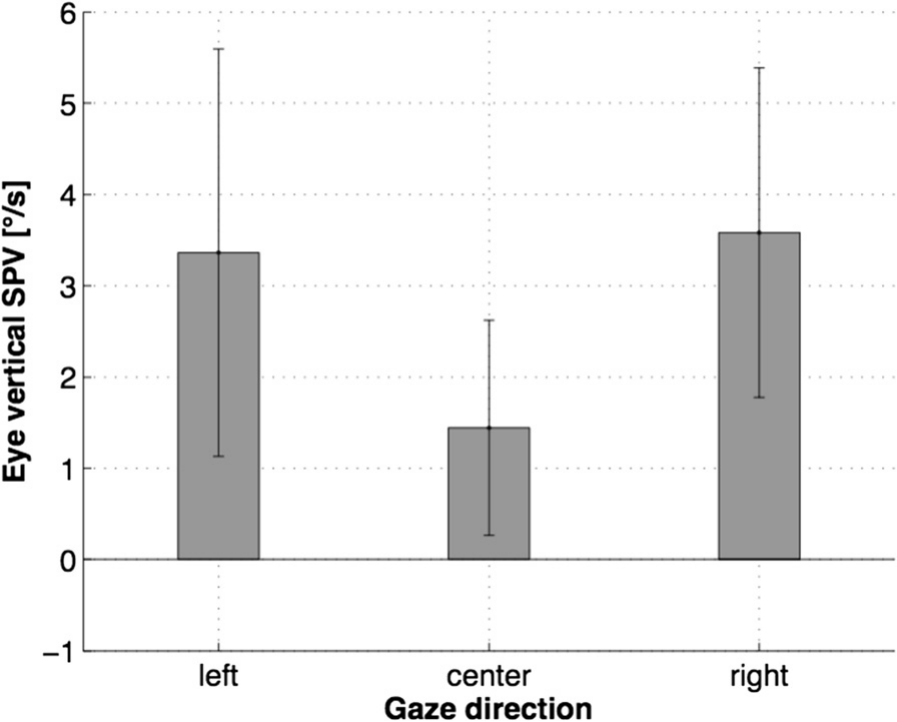
Slow phase velocity (SPV) of the DBN during gaze straight ahead as well as sideways (both left and right 20° off center) for all ten subjects. In gaze straight ahead, the mean SPV is 1.4°/s and increases noticeably in left and right position (mean left 3.36°/s; mean right 3.58°/s)

The mean slow phase velocity (SPV) was moderate during gaze straight ahead (1.44°/s, SD +/- 1.18°/s). As one would expect, SPV increased significantly in sideward gaze (Fig. 2; mean left 3.36°/s, SD +/- 2.23°/s; mean right 3.58°/s, SD +/- 1.8°/s; one-way ANOVA center-left *p* = 0.027, center-right *p* = 0.005). VA in center gaze during static and feedback-driven conditions remained stable throughout both measurements, i.e. revealing no significant change in either condition as the absolute values remained equal. In sideward gaze during the static condition, VA decreased compared to the VA in center gaze in seven out of ten subjects (mean VA, SD: center gaze 0.35 +/- 0.09; left 0.31 +/- 0.09; right 0.28 +/- 0.08; descriptive differences). VA remained equal in center gaze and improved in five out of ten subjects in sideward gaze during the feedback-driven condition compared to the static condition (mean VA, SD: center gaze 0.35 +/- 0.09; left 0.32 +/- 0.09; right 0.33 +/- 0.1; descriptive differences).

**Fig. 3:**
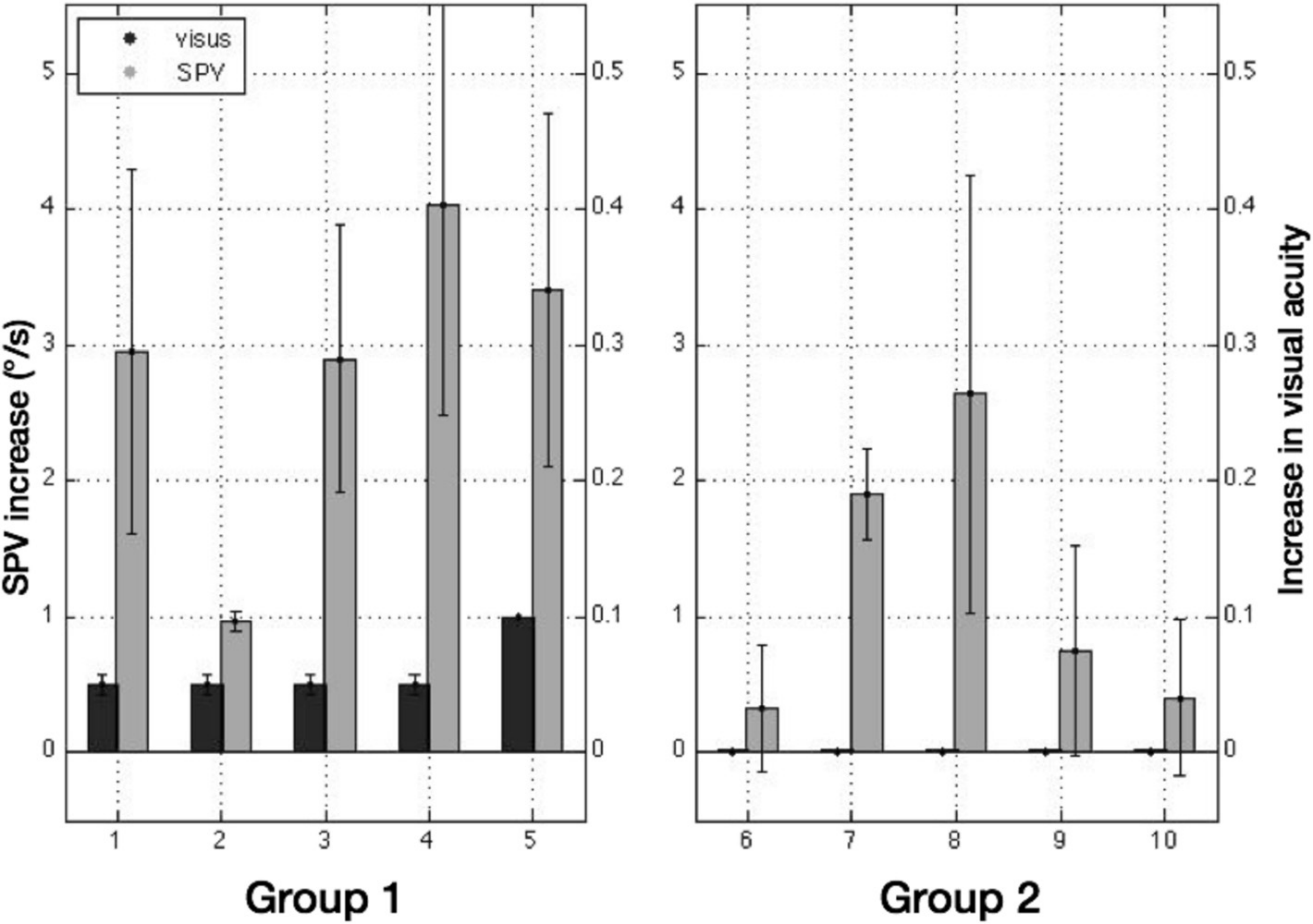
Difference in SPV (grey column) and visual acuity (VA, black column) after allocation in either group 1 (improvement of VA) or group 2 (no improvement in VA)

**Fig. 4:**
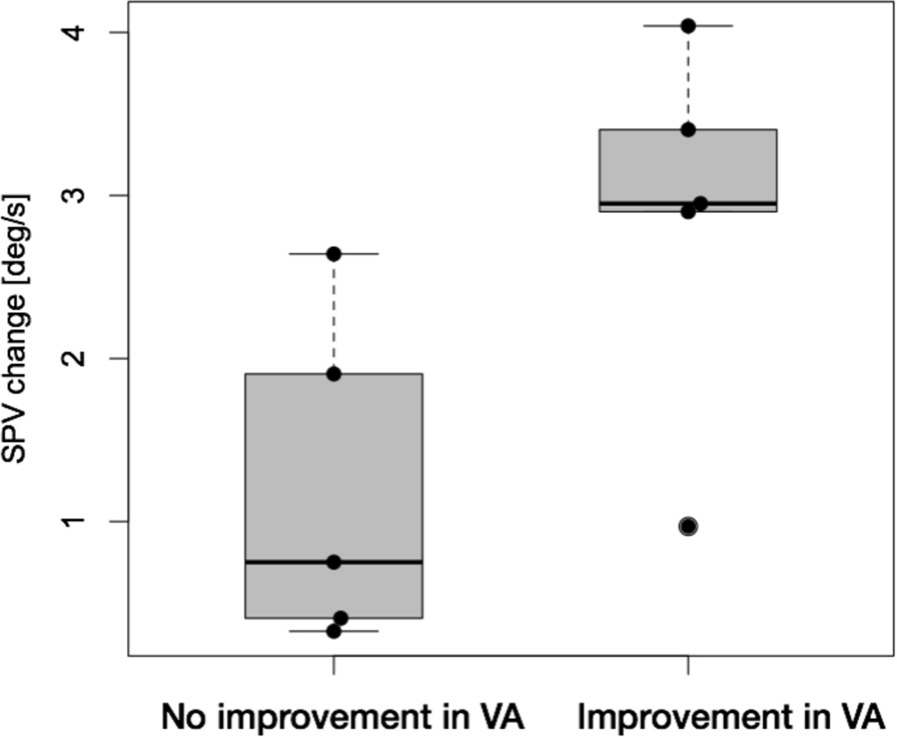
Statistical analysis (ANOVA, *p* = 0.043) shows a significant increase in SPV in the VA improvement group (mean SPV 2.9°/s; each dot indicates one subject) compared to the subjects without VA improvement (mean SPV 1.2°/s)

Comparison of gaze induced changes in DBN correlated strongly with improvements in VA during the feedback-driven condition (r = 0.73, *p* = 0.016), classifing two distinct patient groups. Those patients with DBN whose VA improved during the feedback-driven condition showed the most prominent increase of SPV during sideward gaze (Fig. 3; group 1) compared to those patients (group 2) who did not. The mean improvement of VA was 0.01 in leftward and 0.1 in rightward gaze. In order to compare both groups with inferential statistics, we first tested whether the assumption of normal distribution was fulfilled. According to Shapiro-Wilk test, the data were normally distributed using the VA as dependent variable (group 1: *p* = 0.343; group 2: *p* = 0.251). The subsequent one-way ANOVA showed a significant difference (Fig. 4; *p* = 0.043; dependent variable SPV increase, independent variable feedback condition).

## Discussion

This study investigated the effect of real-time visual feedback using a computer-assisted device on VA in ten patients with DBN syndrome. The major findings of this study were as follows: first, there was no effect on VA during gaze straight ahead, probably due to a low baseline SPV. This applied to patients with high or low SPV in gaze straight ahead. There were four patients with a SPV >1°/s, however there was also no improvement after giving visual feedback in gaze straight ahead. Second, in sideward gaze however, VA improved significantly. If there was a decrease in VA in the static condition in sideward gaze compared to gaze straight ahead, the loss of VA could be compensated by the dynamic visual feedback. If there was no decrease in VA during sideward gaze, there was no improvement in VA accordingly.

Looking at the difference in SPV between gaze straight ahead and in sideward gaze, there is no striking difference in VA during the static condition.

The reason might be a central compensation mechanism for an unstable visual perception due to oscillopsia, which prevents a strong decline in VA. The central compensation mechanism could be further addressed by long-term analysis of acute lesions leading to visual impairment due to nystagmus because of a stroke or in multiple sclerosis. A sudden decrease in VA due to an acute lesion leading to an unstable visual field could reveal the effectiveness of our setup. However, visual impairment and difficulties in reading texts reduce the quality of life and even the capability to work, especially in an increasingly computerized working environment.

A further problem may consist of technical reasons. A stable visual field is inherently necessary for an adequate VA. As there is a slight offset when the eye jumps back on target after the downward saccade, another corrective saccade is needed to refixate the target, if the latter does not match the previous retinal image. As mentioned above, the Landolt C was shown in a predefined area of 7.5° to limit the ocular offset. However, it cannot be ruled out that there occurred a retinal error fostering an unstable perception and therefore limiting the effectiveness of the feedback compensation.

Effects of the positive influence of retinal image stabilization on VA have been previously reported [9, 10]. Yet, our setup presents a different technical approach, as there is no need for a magnetic search coil or the application of an optical device including contact lenses. The results of the previous studies cannot be compared directly to our computer-driven visual feedback due to the different technical approach. However, we can confirm the finding that retinal image stabilization in patients with acquired nystagmus leads to an improved VA. The focus of our study was on expanding the usage of an already existing eye tracking device as an easy-to-use and non-invasive application for an improved VA for reading on a computer screen.

Looking at the results of a study investigating the effect of the drug 4-aminopyridine on VA in DBN syndrome, we can find an improvement of mean VA of 0.07 (from 0.59 to 0.66) in gaze straight ahead with a responder rate of 57 %, i.e. 57 % of patients responded successfully to treatment with 4-aminopyridine by reducing the nystagmus [4]. Therefore, we can conclude that a reduction of SPV actually improves the VA. In our study, we found an improvement of mean VA of 0.1 in rightward gaze and 0.01 in leftward gaze for patients with a significant increase of SPV in sideward gaze. Since SPV in sideward gaze was not analyzed in the study with 4-aminopyridinne^3^ those results cannot be transferred to our study. In the 4-aminopyridine study, the DBN patients showed a higher SPV at baseline than our cohort. Hence, we hypothesize that we did not find an improvement in VA during center gaze due to the small SPV at baseline.

The question arises why there is only a subtle effect in gaze straight ahead using our visual feedback device. As already mentioned above, the reason could be that a central compensation mechanism already compensated for the oscillopsia, which persisted for years (mean duration of symptoms 6.3 years). Furthermore, the reason could be technical. It is possible that the lack of stability of the visual field could explain the poor VA in our experimental setup as well as the minor improvements. However, an even more unstable perception should even further decrease the VA.

Given our findings and other studies using 4-aminopyridine, we can assume that there actually is an improvement in VA after reduction of SPV, as the VA correlated with the improvement of SPV.

For investigating our experimental setup and proof of concept, our cohort can be considered sufficient. Further studies are needed to investigate these findings in a larger patient group.

## Conclusions

This study provides proof of concept that real-time computer-based visual feedback compensates for the SPV in DBN and therefore improves VA. So far, our findings are limited to the mere testing of VA using the Landolt C in patients with chronic DBN. Further studies will have to investigate our results in acute lesions and larger patient cohorts. The novelty of our research was the different technical approach by application of a non-invasive setup using the EyeSeeCam**®** eye tracking system. Recent technological advances in the area of virtual reality displays might soon render this approach feasible in fully mobile settings.

## Abbreviations

ANOVA: analysis of variance
CANVAS: cerebellar ataxia, neuropathy, vestibular areflexia syndrome
CC: cum correctione
DBN: downbeat nystagmus
SC: sin correctione
SD: standard deviation
SPV: slow phase velocity
VA: visual acuity

## References

[1] Strupp M, Thurtell MJ, Shaikh AG, Brandt T, Zee DS, Leigh RJ (2011). Pharmacotherapy of vestibular and ocular motor disorders, including nystagmus. J Neurol.

[2] Tilikete C, Vighetto A (2011). Oscillopsia: causes and management. Curr Opin Neurol.

[3] Bremmer F, Kubischik M, Hoffmann KP, Krekelberg B (2009). Neural dynamics of saccadic suppression. J Neurosci.

[4] Claassen J, Spiegel R, Kalla R, Faldon M, Kennard C, Danchaivijitr C (2013). A randomised double-blind, cross-over trial of 4-aminopyridine for downbeat nystagmus--effects on slowphase eye velocity, postural stability, locomotion and symptoms. J Neurol Neurosurg Psychiatry.

[5] Kalla R, Glasauer S, Buttner U, Brandt T, Strupp M (2007). 4-aminopyridine restores vertical and horizontal neural integrator function in downbeat nystagmus. Brain.

[6] Kalla R, Glasauer S, Schautzer F, Lehnen N, Buttner U, Strupp M (2004). 4-aminopyridine improves downbeat nystagmus, smooth pursuit, and VOR gain. Neurology.

[7] Strupp M, Schuler O, Krafczyk S, Jahn K, Schautzer F, Buttner U (2003). Treatment of downbeat nystagmus with 3,4-diaminopyridine: a placebo-controlled study. Neurology.

[8] Schneider E, Villgrattner T, Vockeroth J, Bartl K, Kohlbecher S, Bardins S (2009). EyeSeeCam: an eye movement-driven head camera for the examination of natural visual exploration. Ann N Y Acad Sci.

[9] Leigh RJ, Rushton DN, Thurston SE, Hertle RW, Yaniglos SS (1988). Effects of retinal image stabilization in acquired nystagmus due to neurologic disease. Neurology.

[10] Yaniglos SS, Leigh RJ (1992). Refinement of an optical device that stabilizes vision in patients with nystagmus. Optom Vis Sci.

[11] Ricci F, Cedrone C, Cerulli L (1998). Standardized measurement of visual acuity. Ophthalmic Epidemiol.

